# Ventriculoperitoneal shunt versus transverse sinus stenting in idiopathic intracranial hypertension: A systematic review and meta-analysis

**DOI:** 10.1016/j.bas.2026.106264

**Published:** 2026-08-07

**Authors:** Badr Hafiz, Alaa Turkistani, Waseem Alharthi, Tala Alsindi, Retaj Faisal Arif, Mohammed Binmahfoodh, Mohammed Aref

**Affiliations:** a Department of Neurosciences, King Faisal Specialist Hospital and Research Centre, Jeddah, Saudi Arabia; b College of Medicine, Taif University, Taif, Saudi Arabia; c Neurosurgery Section, Department of Surgery, King Abdulaziz University Hospital, Jeddah, Saudi Arabia; d Department of Surgery, Royal College of Surgeons, Dublin, Ireland; e Faculty of Medicine, Alfaisal University, Riyadh, Saudi Arabia

**Keywords:** Cerebrospinal fluid shunts, Endovascular procedures, Headache disorders, Idiopathic intracranial hypertension, Ventriculoperitoneal shunt

## Abstract

**Introduction:**

Idiopathic intracranial hypertension (IIH) is characterized by elevated intracranial pressure with risk of visual impairment. Ventriculoperitoneal shunting (VPS) and transverse sinus stenting (TSS) are commonly used surgical options, but comparative evidence remains limited.

**Methods:**

This systematic review and meta-analysis followed PRISMA guidelines and was registered in PROSPERO (CRD420251050247). PubMed, Web of Science, Cochrane CENTRAL, Embase, and Scopus were searched (2000–2025). Adult IIH patients treated with VPS or TSS were included. Random-effects meta-analysis was performed.

**Results:**

Seventeen studies were included. Intracranial pressure reduction was significant following TSS (SMD −4.58; 95% CI: −5.72 to −3.44; *p* < 0.0001); however, no pooled VPS data were available, precluding direct comparison. Visual improvement occurred in 83.9% of patients, with no significant difference between TSS (84.1%) and VPS (83.3%) (*p* = 0.933). Headache improvement was observed in 73.6%, with no significant between-group difference (*p* = 0.628). Complication rates were low overall (0.1%), although the higher reported rate with TSS (1.56%) than VPS (approximately 0%) most likely reflects inconsistent complication definitions and incomplete event capture rather than a true procedural risk difference. Revision rates were lower with TSS (9.8%) than VPS (24.5%), but the difference was not statistically significant (*p* = 0.305).

**Conclusion:**

VPS and TSS provide comparable clinical outcomes in IIH. TSS demonstrated numerically lower revision rates, although without statistical significance. These findings should be interpreted cautiously because the available evidence is derived predominantly from non-randomized, indirect comparisons with substantial indication bias, and the treatment groups likely differed systematically at baseline. Prospective phenotype-guided comparative studies are needed to better define the optimal surgical strategy.

## Highlights

• Systematic review and meta-analysis comparing ventriculoperitoneal shunting and transverse sinus stenting for IIH patients.
• Seventeen studies were included, demonstrating comparable visual and headache outcomes between interventions.
• Intracranial pressure reduction was confirmed following transverse sinus stenting; no pooled VP shunt comparison was available.
• Transverse sinus stenting showed numerically lower revision rates, but the difference was not statistically significant.
• Current evidence is limited by indirect comparisons, indication bias, and heterogeneous studies, supporting individualized treatment.

## 1 Introduction

Idiopathic intracranial hypertension (IIH), also known as pseudotumor cerebri, is a neurological condition that is marked by persistently high intracranial pressure (ICP) without a structural lesion, hydrocephalus, or venous thrombosis (Wall, 2010). Even though the incidence of IIH is comparatively low, the condition presents a significant clinical burden associated with the likelihood of profound visual impairment and long-term disabling headaches. Epidemiological studies indicate that the incidence of IIH is on the rise by an average of 0.5-2 per 100,000 people annually in the general population, although in high-risk groups such as obese women of reproductive age, the incidence is very high with an annual rate of almost 22 per 100,000 (Kilgore et al., 2017). The symptoms typically include headache, transient visual obscurations, papilledema, diplopia, and pulsatile tinnitus, which are the result of prolonged increases in ICP and impaired cerebrospinal fluid (CSF) dynamics (Wall, 2010). The continuous increase in ICP can cause a gradual injury to the optic nerve and irreversible loss of vision, so timely diagnosis and therapy are essential to prevent long-term morbidity (Wakerley et al., 2020).

The pathophysiology of IIH has not yet been fully understood and it is believed to be multifactorial. Some of the mechanisms proposed are impaired CSF absorption, disturbed cerebral venous drainage, hormonal and metabolic alterations caused by obesity and elevated venous sinus pressure (Lowe et al., 2025). Recent neuroimaging studies have indicated that transverse venous sinus stenosis is common in patients with IIH, and that venous outflow obstruction could play a central role in the pathogenesis or progression of the disease (Cheng et al., 2024). In fact, recent research has documented the phenomenon of venous sinus stenosis in 10-90% of patients with IIH, based on the imaging modality and choice of diagnostic criteria (Pandey et al., 2024). High venous pressure could decrease the CSF absorption at the arachnoid granulations to create a vicious cycle of elevated ICP and additional venous compression (Zhao et al., 2022). These mechanisms have led to the development of new treatment modalities for the venous system.

Medical treatment is the initial step of IIH management, such as weight loss and pharmacologic therapy, especially carbonic anhydrase inhibitors, e.g., acetazolamide (Naqvi et al., 2025). Although most patients respond to medical treatment with an improvement in their symptoms, a significant percentage of patients develop a medically refractory disease or progressive visual loss that requires surgical treatment. Historically, surgical therapy for refractory IIH has centered on CSF diversion, most commonly in the form of ventriculoperitoneal shunting (VPS), though lumboperitoneal and, less commonly, ventriculoatrial shunting has also historically been used (Kalyvas et al., 2021). This review focuses specifically on VPS as the CSF diversion comparator to TSS, given its predominance in the modern comparative literature. VP shunts operate by diverting the surplus CSF from the ventricles into the peritoneal cavity and decreasing the ICP, thus relieving the symptoms. Nonetheless, these procedures are linked to severe complications, such as shunt failure, infection, excessive drainage, and repeated revision surgeries that can occur in a significant percentage of patients during the long-term period (Fowler et al., 2026). These limitations have provoked attention to other treatment methods that would directly target the underlying venous pathology.

For patients with IIH and proven venous sinus stenosis, venous sinus stenting (VSS) has become increasingly popular. Stenting can also positively influence the resorption of the CSF and decrease ICP by normalizing the venous outflow and reducing the trans-stenotic pressure gradient (Liu et al., 2017). A number of clinical trials have shown good results with the technique. In a systematic review, papilledema was improved or resolved in about 93.7% of patients and headache improved in almost 79.6% of patients after venous sinus stenting (Nicholson et al., 2019). On the same note, combined studies on more than 1000 patients treated revealed that the opening pressure in the CSF dropped significantly and that there were also improvements in symptoms of tinnitus, visual disturbances, and headache (Azzam et al., 2024). Although these are positive outcomes, issues about complications, restenosis, and durability of the intervention in the long run are still present.

There is a lack of comparative data between VSS and CSF diversion procedures. According to the published literature, VSS can lead to symptom improvement in about 77–87% of patients with restenosis or chronic symptoms possible in up to 22% of patients in some studies (Lim et al., 2024; Teleb et al., 2013). The changing trend in IIH clinical practice is favoring VSS rather than conventional CSF shunts because of the available data, which proves higher effectiveness and lower readmission rates and patient outcomes. Studies from 2016 to 2020 reveal that VSS procedures on IIH rose by approximately 80% in a year, whereas the shunting decreased by approximately 19% (Abbasi et al., 2024).

Since the use of VSS is on the rise, and the long history of VPS in the management of refractory IIH, there is still uncertainty regarding the best intervention based on clinical and safety outcomes. Hence, the relative effectiveness and the complication profiles of the available evidence require a detailed synthesis to be clear. The current systematic review is a comparison between VPS and TSS in patients with IIH based on their clinical outcomes (reduction of ICP, alleviation of visual symptoms, alleviation of headache, rate of complication, and necessity to undergo other interventions).

## 2 Materials and methods

### 2.1 Study design and reporting guidelines

This systematic review was conducted in accordance with the Preferred Reporting Items for Systematic Reviews and Meta-Analyses (PRISMA) guidelines (Page et al., 2021). The review protocol was prospectively registered in the PROSPERO international prospective register of systematic reviews (Registration number: CRD420251050247).

### 2.2 Search strategy and information sources

The literature search was conducted comprehensively to find the studies that considered the use of VPS and TSS to treat IIH. Five electronic bibliographic databases were systematically searched: PubMed, Web of Science (WoS), Cochrane Central Register of Controlled Trials (CENTRAL), Embase and Scopus. Searches were done on publications since January 1, 2000, up to May 1, 2025. Only articles published in the English language were included. The search strategy involved the use of combinations of keywords and controlled vocabulary in items associated with IIH, VPS, CSF shunting, TSS and venous sinus stenting. Besides the search of databases, reference lists of the relevant studies were screened manually to retrieve more eligible studies.

### 2.3 Study selection

**Fig. 1:**
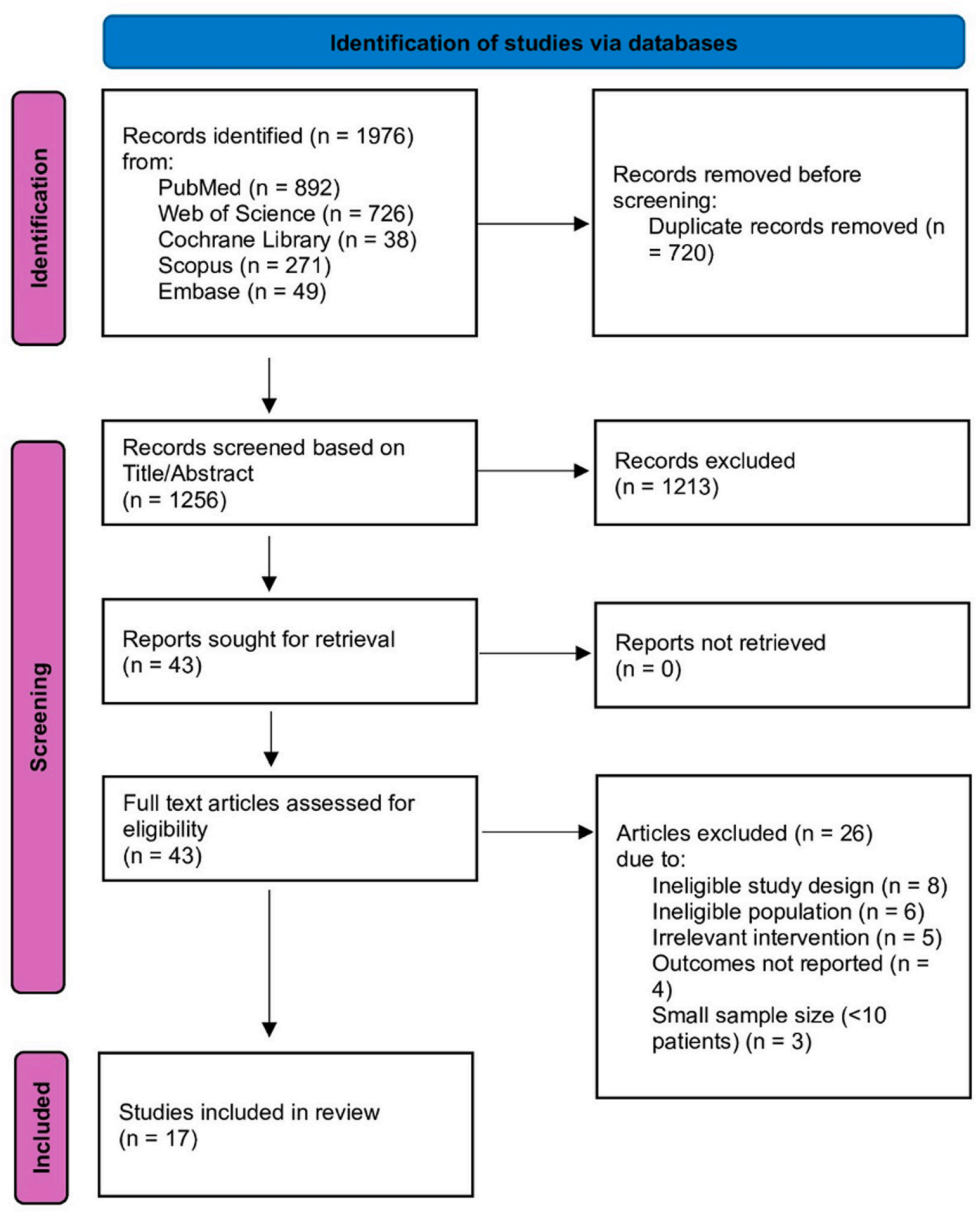
PRISMA chart showing selection and inclusion of studies.

All identified records were exported into reference management software and screened using Rayyan (https://rayyan.qcri.org/). A total of 1976 records were initially identified across the five databases (PubMed = 892, Web of Science = 726, Cochrane = 38, and Scopus = 271 and Embase = 49). After removing 720 duplicate records, 1256 unique studies remained for title and abstract screening. Two reviewers independently screened these records against predefined eligibility criteria. During this stage, 1213 records were excluded based on irrelevance to the study topic, leaving 43 studies for full-text review. The full texts of these articles were assessed for eligibility, and 26 studies were excluded due to reasons such as inappropriate study design, insufficient outcome reporting, or non-eligible populations. Ultimately, 17 studies met the inclusion criteria and were included in this review (Fig. 1). Disagreements among reviewers in the screening were resolved through discussion and reaching a consensus.

### 2.4 Eligibility criteria

Studies were considered eligible if they included adult patients (18 years or older) diagnosed with IIH who underwent either VPS or TSS as a therapeutic intervention. For the purposes of this review, VPS was defined as any ventricular-to-peritoneal CSF diversion procedure; studies in which the comparator arm was reported only as generic “CSF shunting” or “CSF diversion” without specifying the diversion route were included only where supplementary data confirmed a ventriculoperitoneal approach. Studies evaluating lumboperitoneal or ventriculoatrial shunting as a distinct, separately reported comparator were excluded from the VPS pooled estimate to preserve intervention specificity. Randomized controlled trials, prospective and retrospective cohort studies, case–control studies and case series involving more than 10 patients were included. Both comparative and non-comparative studies were eligible provided they reported relevant clinical outcomes related to either intervention. Studies were excluded if they involved pediatric populations, secondary intracranial hypertension due to identifiable causes (such as venous sinus thrombosis or intracranial tumors), animal or cadaveric models, case reports, small case series with fewer than 10 patients, conference abstracts lacking full data, editorials, letters, or narrative reviews.

### 2.5 Data extraction

Two reviewers independently extracted the data with the use of a standardized data extraction form. The information obtained included study characteristics (authors, year of publication, country, and study design), patient demographics, sample size, type of intervention (VPS or TSS), and reported clinical outcomes. Primary outcomes included reduction in ICP, improvement or resolution of visual symptoms, and headache improvement. Secondary outcomes included complication rates, re-intervention rates, quality of life measures, mortality, hospital readmissions, and duration of hospitalization. Inconsistencies in the extracted data were resolved through discussion and agreement between the reviewers.

“For consistency throughout this manuscript, the terms *transverse sinus stenting (TSS)* and *venous sinus stenting (VSS)* are used interchangeably to refer to venous sinus stenting procedures. Likewise, *ventriculoperitoneal shunting (VPS)* refers exclusively to ventriculoperitoneal cerebrospinal fluid (CSF) diversion, and the broader terms *CSF diversion* or *CSF shunting* are used only when reflecting the terminology reported in the original studies."

### 2.6 Quality assessment (risk of bias)

**Fig. 2:**
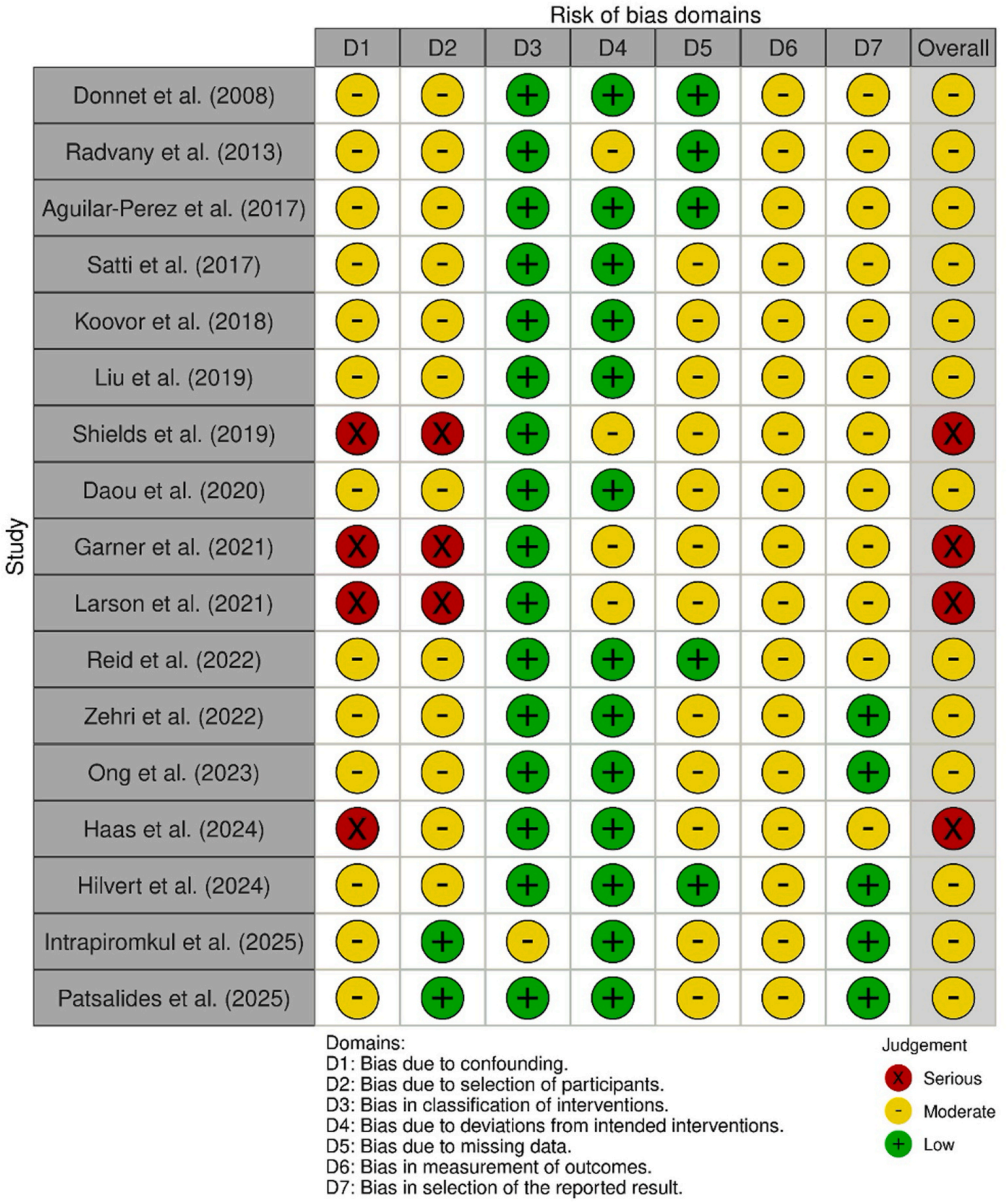
Risk of bias assessment of non-randomized studies using ROBINS-I: Traffic light plot.

**Fig. 3:**
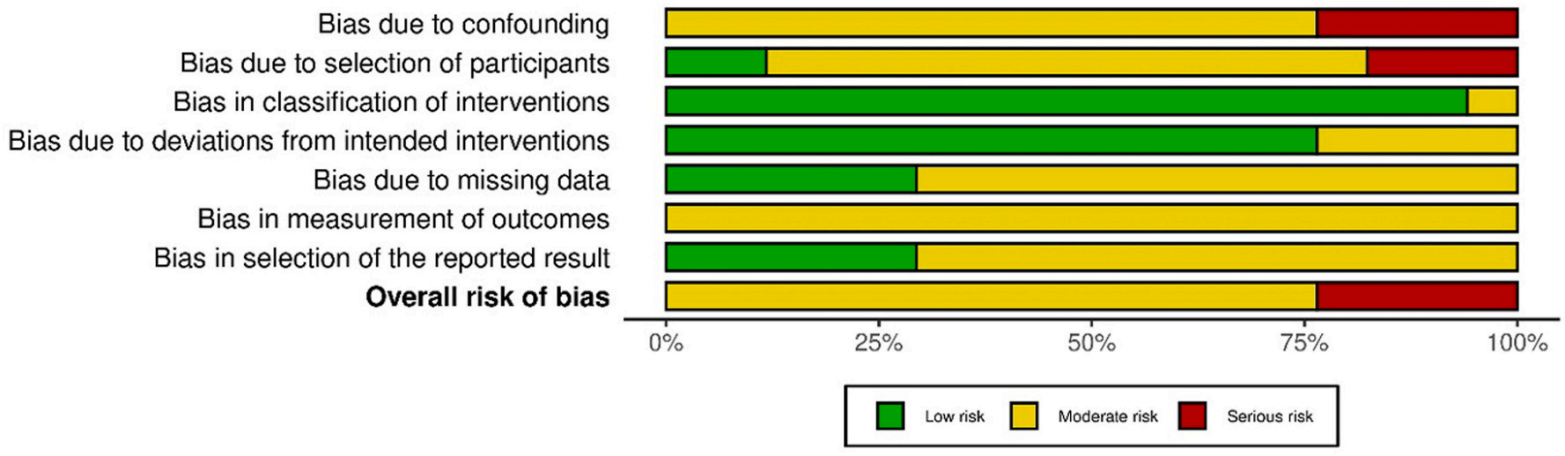
Summary of risk of bias judgments for non-randomized studies (ROBINS-I).

The methodological quality of the included studies was evaluated using the ROBINS-I tool for non-randomized studies. The studies included showed a low to moderate risk of bias with the most frequently encountered issues being related to confounding, participant selection, and the retrospective nature of the majority of studies (Fig. 2, Fig. 3).

A moderate risk of confounding was observed across the majority of studies, including Donnet et al. (2008) (Donnet et al., 2008), Radvany et al. (2013) (Radvany et al., 2013), Aguilar-Pérez et al. (2017) (Aguilar-Pérez et al., 2017), Satti et al. (2017) (Satti et al., 2017), Koovor et al. (2018) (Koovor et al., 2018), Liu et al. (2019) (Liu et al., 2019), Shields et al. (2019) (Shields et al., 2019), Daou et al. (2020) (Daou et al., 2020), Garner et al. (2021) (Garner et al., 2021), Larson et al. (2021) (Larson et al., 2021), Reid et al. (2022) (Reid et al., 2022), Zehri et al. (2022) (Zehri et al., 2022), Ong et al. (2023) (Ong et al., 2023), Haas et al. (2024) (Haas et al., 2024), Hilvert et al. (2024) (Hilvert et al., 2024), and Intrapiromkul et al. (2025) (Intrapiromkul et al., 2025). This was mainly because of the absence of randomization and failure to fully correct baseline differences in the severity of intracranial hypertension, symptoms duration, previous medical treatment and comorbidity (e.g., obesity). All these factors could have affected treatment allocation, as well as clinical outcomes.

The rating of selection bias was low to moderate. The adult patients with IIH undergoing VPS or TSS were well defined populations in most of the studies. Nonetheless, some of the studies, especially retrospective case series and single-center cohorts, faced the risk of selection bias because of non-consecutive recruitment, small sample size, or highly selected patients with refractory disease. Intrapiromkul et al. (2025) (Intrapiromkul et al., 2025), showed a smaller risk in this area because of the larger population inclusion.

The risk of bias associated with the categorization of interventions in all the studies was regarded as low because the interventions (VPS and TSS) were well-defined, standardized, and applied consistently. On the same note, errors in intended interventions were low with majority of the studies registering outcomes that were based on the original treatment plan with no major crossover or protocol error.

The risk of bias because of missing information was normally minimal, with the majority of studies reporting sufficient follow-up and result information. Nevertheless, not all studies fully reported long-term follow-up results or attrition rates especially in retrospective studies, which can cause a certain degree of uncertainty in outcome measurement.

There was general low outcome measurement bias. The objective clinical or radiological measurement of vital outcomes like ICP, papilledema resolution and visual acuity were evaluated. However, subjective outcomes like improvement of headache and quality of life are likely to be subject to reporting bias, however, a number of studies used validated instruments (e.g., HIT-6, WHOQOL-BREF), which increases reliability.

The bias in reporting was low to moderate. Although the majority of studies presented primary clinical outcomes, outcome definitions were heterogeneous, and not all studies reported complications and re-intervention rates.

### 2.7 Statistical plan

A random-effects model was used as a meta-analysis to explain between-study variation. Continuous outcomes (ICP) were analyzed using standardized mean difference (Hedges' g), while dichotomous outcomes (visual improvement, headache improvement, complications, and revision rates) were pooled as proportions with 95% confidence intervals. The inverse variance method with restricted maximum-likelihood estimation was applied. Heterogeneity was assessed using the I^2^ statistic and Cochran's Q test. Subgroup analyses comparing TSS and VPS were performed. Publication bias was evaluated using funnel plot asymmetry. A p-value <0.05 was considered statistically significant.

## 3 Results

This systematic review involved the inclusion of 17 studies. The studies were a heterogeneous collection of prospective and retrospective designs, and most were retrospective cohort and case series. Sample size was quite diverse (small cohorts of 10 patients and large population-based samples of more than 2000 patients). All the studies were dominated by females whose proportions exceeded 80% indicating the established epidemiological trend of IIH. The average or median age of the participants was usually between early 30s to the early 40s, which confirms that the condition mostly affects young and middle-aged adults. Body mass index, when mentioned, was always high with the majority of reports showing mean BMI values in the obese category, which further corroborates obesity as a significant risk factor (Table 1).

**Table 1:** Baseline characteristics of included studies evaluating ventriculoperitoneal shunting and transverse sinus stenting.

First Author (Year)	Study Design	Sample Size (n)	Age (Mean/Median, Range/SD)	Female (%)	BMI (Mean/Range)
Donnet et al. (2008) (Donnet et al., 2008)	Prospective case series	10	41.8 (28–60)	80%	22–37 kg/m^2^
Radvany et al. (2013) (Radvany et al., 2013)	Case series	12	39 (21–55)	91.7%	32.6 kg/m^2^
Aguilar-Pérez et al. (2017) (Aguilar-Pérez et al., 2017)	Retrospective cohort	51	40 (5–66)	80.4%	N/A
Satti et al. (2017) (Satti et al., 2017)	Retrospective cohort	43	34.9 (21–54)	90.7%	34.8 (21–56) kg/m^2^
Koovor et al. (2018) (Koovor et al., 2018)	Retrospective case series	16	35	93.8%	40.65 kg/m^2^
Liu et al. (2019) (Liu et al., 2019)	Retrospective observational	88	39.01 ± 9.73 (18–60)	76.1%	26.75 ± 3.79 kg/m^2^
Shields et al. (2019) (Shields et al., 2019)	Retrospective observational	42	32.5 (15–52)	90.5%	35.6 kg/m^2^
Daou et al. (2020) (Daou et al., 2020)	Retrospective cohort	163	32.6 ± 8.8	92%	37.9 ± 8.3 kg/m^2^
Garner et al. (2021) (Garner et al., 2021)	Retrospective case series	81	35.9	90.1%	37.6 kg/m^2^
Larson et al. (2021) (Larson et al., 2021)	Retrospective case series	16	36.4 ± 10.4	100%	35.6 ± 7.5 kg/m^2^
Reid et al. (2022) (Reid et al., 2022)	Retrospective cohort	32	31.2 (17–46)	84.4%	N/A
Zehri et al. (2022) (Zehri et al., 2022)	Retrospective case series	10	31 (19–37)	90%	41.2 (24.8–57.5) kg/m^2^
Ong et al. (2023) (Ong et al., 2023)	Retrospective cohort	83	34.9	90.4%	N/A
Haas et al. (2024) (Haas et al., 2024)	Retrospective case series	25	42	88%	N/A
Hilvert et al. (2024) (Hilvert et al., 2024)	Case–control study	87	33 (VSS); 36 (VPS)	96% (VSS); 90% (VPS)	36 (VSS); 40 (VPS) kg/m^2^
Intrapiromkul et al. (2025) (Intrapiromkul et al., 2025)	Retrospective cohort	2450	37.1 ± 11.5 (VSS); 36.8 ± 13.4 (CSF shunt)	89.9% (VSS); 91.7% (CSF shunt)	34.7 ± 10.3 (VSS); 32.7 ± 9.1 (CSF shunt) kg/m^2^
Patsalides et al. (2025) (Patsalides et al., 2025)	Prospective trial	39	35.7 ± 12 (19–65)	97.4%	N/A

Abbreviations: BMI: Body Mass Index; SD: Standard Deviation; N/A: Not Available; VSS: Venous Sinus Stenting; VPS: Ventriculoperitoneal Shunt; CSF: Cerebrospinal Fluid.

The studies incorporated showed significant differences in the nature of intervention as well as the period of follow up. Most of the studies considered transverse or venous sinus stenting, which is a growing trend in minimally invasive practice, and few studies were on VPS. Where a study's comparator arm was reported in the original publication as “CSF shunt” rather than as “VPS” (e.g., Intrapiromkul et al., 2025 (Intrapiromkul et al., 2025)), this was confirmed to represent VPS specifically before inclusion in the VPS pooled estimate; this terminology is retained in (Table 1) for fidelity to the source publication. There was wide variation in the duration of follow-up in studies with short term follow-up of up to 30 days and long term follow-up up to more than 4 years. The majority of the studies mentioned the follow-up periods of 6 to 36 months, which is a fair time range of assessing intermediate clinical outcomes (i.e., symptom improvement and complication rates). Some of the studies included a combination of clinical and imaging follow-up, which points to the differences in the use of outcome monitoring methods. Nevertheless, there were inconsistencies in the timing and reporting of follow up duration and methodology with some studies not providing specific times or only end points (Table 2).

**Table 2:** Intervention type and follow-up duration of included studies.

First Author (Year)	Intervention	Follow-up Duration
Donnet et al. (2008) (Donnet et al., 2008)	Transverse sinus stenting	Mean 17 ± 10.1 months (Pandey et al., 2024; Zhao et al., 2022; Naqvi et al., 2025; Kalyvas et al., 2021; Fowler et al., 2026; Liu et al., 2017, 2019; Nicholson et al., 2019; Azzam et al., 2024; Lim et al., 2024; Teleb et al., 2013; Abbasi et al., 2024; Page et al., 2021; Donnet et al., 2008; Radvany et al., 2013; Aguilar-Pérez et al., 2017; Satti et al., 2017; Koovor et al., 2018; Shields et al., 2019; Daou et al., 2020; Garner et al., 2021; Larson et al., 2021; Reid et al., 2022; Zehri et al., 2022; Ong et al., 2023; Haas et al., 2024; Hilvert et al., 2024; Intrapiromkul et al., 2025; Patsalides et al., 2025; Fargen et al., 2023; Fargen, 2022)
Radvany et al. (2013) (Radvany et al., 2013)	Transverse sinus stenting	Mean 16 months (Kalyvas et al., 2021; Fowler et al., 2026; Liu et al., 2017, 2019; Nicholson et al., 2019; Azzam et al., 2024; Lim et al., 2024; Teleb et al., 2013; Abbasi et al., 2024; Page et al., 2021; Donnet et al., 2008; Radvany et al., 2013; Aguilar-Pérez et al., 2017; Satti et al., 2017; Koovor et al., 2018; Shields et al., 2019; Daou et al., 2020; Garner et al., 2021; Larson et al., 2021; Reid et al., 2022; Zehri et al., 2022; Ong et al., 2023; Haas et al., 2024; Hilvert et al., 2024; Intrapiromkul et al., 2025; Patsalides et al., 2025; Fargen et al., 2023; Fargen, 2022)
Aguilar-Pérez et al. (2017) (Aguilar-Pérez et al., 2017)	Transverse sinus stenting	Median 49 months
Satti et al. (2017) (Satti et al., 2017)	Transverse sinus stenting	Mean 13.5 months (1–63)
Koovor et al. (2018) (Koovor et al., 2018)	Transverse sinus stenting	Mean 35.5 months (clinical); imaging 10.3 months
Liu et al. (2019) (Liu et al., 2019)	Transverse sinus stenting	Follow-up until July 2017
Shields et al. (2019) (Shields et al., 2019)	Transverse sinus stenting	Mean 25.6 months (8.7–60.7)
Daou et al. (2020) (Daou et al., 2020)	Ventriculoperitoneal shunt	29.6 ± 35.5 months
Garner et al. (2021) (Garner et al., 2021)	Transverse sinus stenting	Mean 10 months (0.5–38)
Larson et al. (2021) (Larson et al., 2021)	Transverse sinus stenting	Clinical: 7 ± 5.4 months; Imaging: 3.6 ± 2.2 months
Reid et al. (2022) (Reid et al., 2022)	Transverse sinus stenting	Mean 19 months (0–56)
Zehri et al. (2022) (Zehri et al., 2022)	Venous sinus stenting	Mean 60.5 weeks (6–112)
Ong et al. (2023) (Ong et al., 2023)	Venous sinus stenting	Not reported
Haas et al. (2024) (Haas et al., 2024)	Venous sinus stenting	30 days
Hilvert et al. (2024) (Hilvert et al., 2024)	Venous sinus stenting	376 days
Intrapiromkul et al. (2025) (Intrapiromkul et al., 2025)	Transverse sinus stenting	12 months
Patsalides et al. (2025) (Patsalides et al., 2025)	Transverse sinus stenting	12 months

Abbreviations: CT: Computed Tomography; N/A: Not Available.

Clinical outcome analysis showed that both ventriculoperitoneal shunts and venous sinus stents were correlated with a significant improvement in various domains with ICP decreases of about 9.1 cmH_2_O to more than 24 cmH_2_O, and percentage decreases reaching 75-92% in certain studies. The normal ICP was usually reported to be between 32.9 and 41.7 cmH_2_O, but fell as low as 16-21.5 cmH_2_O, after intervention. There were significant visual improvements as visual outcomes improved or papilledema improved or resolved in approximately 74-100% of patients and general visual symptom improvement of 80-97%. The residual visual disturbances were lower in venous sinus stenting groups (22.9) as compared to CSF shunting (32.3). In patients, the results of headaches were improved in 55.6 to 92.5% with full recovery being maintained in up to 78.3% of stenting patients as opposed to 34.9% of shunted patients. The complication rates were mostly low with major complications of 0-8.1% with certain events like in-stent thrombosis recorded in about 2.4%. In 6-7% of the procedures, minor or access-related complications were reported. There were no differences in the percentage of re-intervention or revision but it was significantly low in venous sinus stenting with the failure of the treatment being noted in 9.6% as opposed to 38.6% in the case of cerebral spinal fluid shunting. The measures of quality of life were also improved, and standardized scores went up by about 10-20 points, which included HIT-6 and WHOQOL-BREF. There were few cases of mortality (with 2 deaths reported), in all of the included studies but not always associated with the interventions (Table 3).

**Table 3:** Clinical outcomes, complications, and Re-intervention rates in included studies.

First Author (Year)	ICP Change	Visual Outcomes	Headache Outcomes	Complications	Re-intervention	QoL Outcomes	Mortality
Donnet et al. (2008) (Donnet et al., 2008)	↓ 40.2 → 16 cmH_2_O	100% papilledema resolution	Majority improved/resolved	No major	10%	N/A	0
Radvany et al. (2013) (Radvany et al., 2013)	N/A	83% improved	Majority improved	0% serious	25%	N/A	0
Aguilar-Pérez et al. (2017) (Aguilar-Pérez et al., 2017)	N/A	81.5% improved	84% improved	Minor 6–7%	15.7%	N/A	0
Satti et al. (2017) (Satti et al., 2017)	Mean 35.8 mmHg	Mixed improvement	69% improved	0%	4.7%	N/A	N/A
Koovor et al. (2018) (Koovor et al., 2018)	↓ from 408 mmH_2_O	100% resolution	Majority improved	0%	0%	N/A	0
Liu et al. (2019) (Liu et al., 2019)	↓ significant	84% improved	92.5% improved	0%	N/A	N/A	2 deaths
Shields et al. (2019) (Shields et al., 2019)	Mean 38 cmH_2_O	74% resolution	43% resolution	2.4% thrombosis	15%	N/A	0
Daou et al. (2020) (Daou et al., 2020)	Baseline 41.7 cmH_2_O	∼88% improved	∼58% improved	N/A	N/A	Improved	N/A
Garner et al. (2021) (Garner et al., 2021)	↓ 9.1 cmH_2_O	61% improved	Improved (HIT-6 ↓)	1.2%	25.9%	Improved	0
Larson et al. (2021) (Larson et al., 2021)	N/A	84.6% improved	Minimal persistence	0%	6.25%	N/A	0
Reid et al. (2022) (Reid et al., 2022)	37.1 cmH_2_O	96% resolved	N/A	Minor	6%	Improved	0
Zehri et al. (2022) (Zehri et al., 2022)	38.2 cmH_2_O	80% improved	55.6% improved	20%	20%	N/A	0
Ong et al. (2023) (Ong et al., 2023)	N/A	91.6% improved	78.3% resolved	2.9%	8.4%	N/A	0
Haas et al. (2024) (Haas et al., 2024)	↓ 75–92%	Improved	72% improved	0%	N/A	N/A	0
Hilvert et al. (2024) (Hilvert et al., 2024)	36–37 cmH_2_O	95–100% improved	>90% improved	N/A	10%	N/A	N/A
Intrapiromkul et al. (2025) (Intrapiromkul et al., 2025)	N/A	Better in stenting	Better in stenting	N/A	Lower in stenting	N/A	N/A
Patsalides et al. (2025) (Patsalides et al., 2025)	↓ 32.9 → 21.5 cmH_2_O	97% improved	85.3% improved	8.1%	5.1%	Improved	0

**Abbreviations:** CSF: Cerebrospinal Fluid; HIT-6: Headache Impact Test-6; ICP: Intracranial Pressure; N/A: Not Available; QoL: Quality of Life.

It should be noted that the majority of included studies were single-arm observational series evaluating either VPS or TSS in isolation, rather than direct comparative cohorts. As such, the subgroup comparisons presented below reflect indirect comparisons across distinct patient populations rather than head-to-head trial data, and the pooled estimates should be interpreted with this structural limitation in mind.

### 3.1 Meta-analysis of quantitative outcomes

**Fig. 4:**
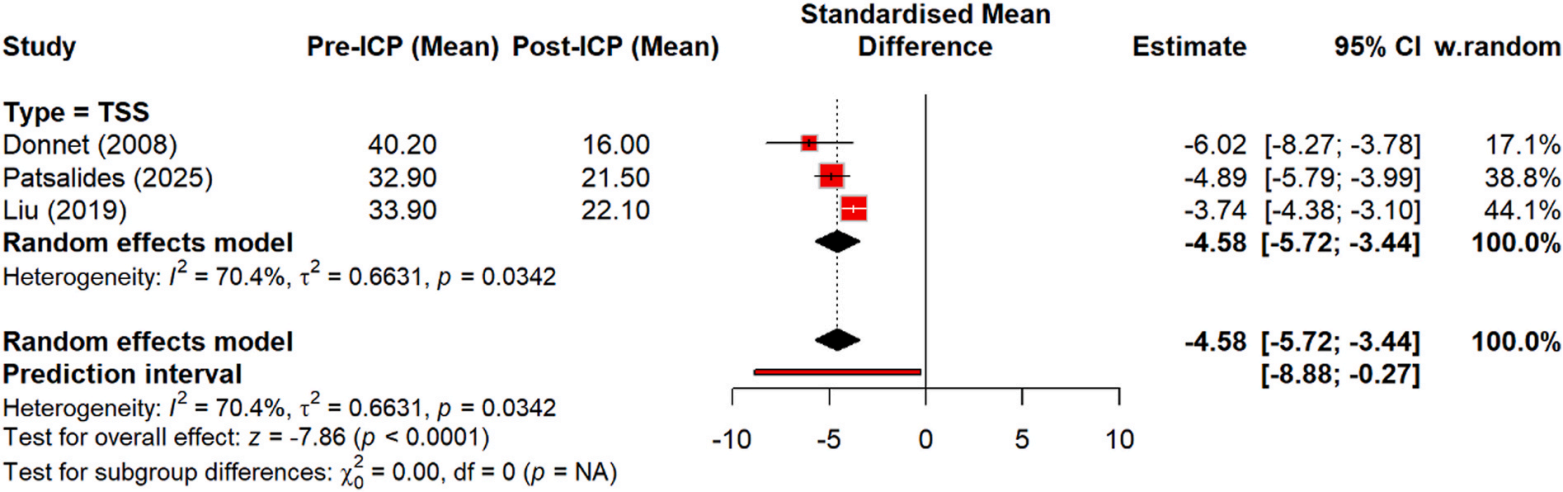
Comparison of ICP between two Interventions.

Our meta-analysis assessed pooled outcomes for VPS and TSS in IIH, where data permitted. For ICP reduction, only TSS studies reported extractable quantitative pre/post data, yielding a large and statistically significant pooled reduction (SMD −4.58; 95% CI: −5.72 to −3.44; p < 0.0001). No comparable pooled VPS estimate was available for this outcome due to inconsistent or non-standardized ICP reporting in VPS studies. Consequently, this result characterizes the magnitude of ICP reduction achieved with TSS alone and does not constitute evidence of superiority over VPS; heterogeneity was also substantial (I^2^ = 70.4%), suggesting variability in study populations or measurement methods (Fig. 4).

**Fig. 5:**
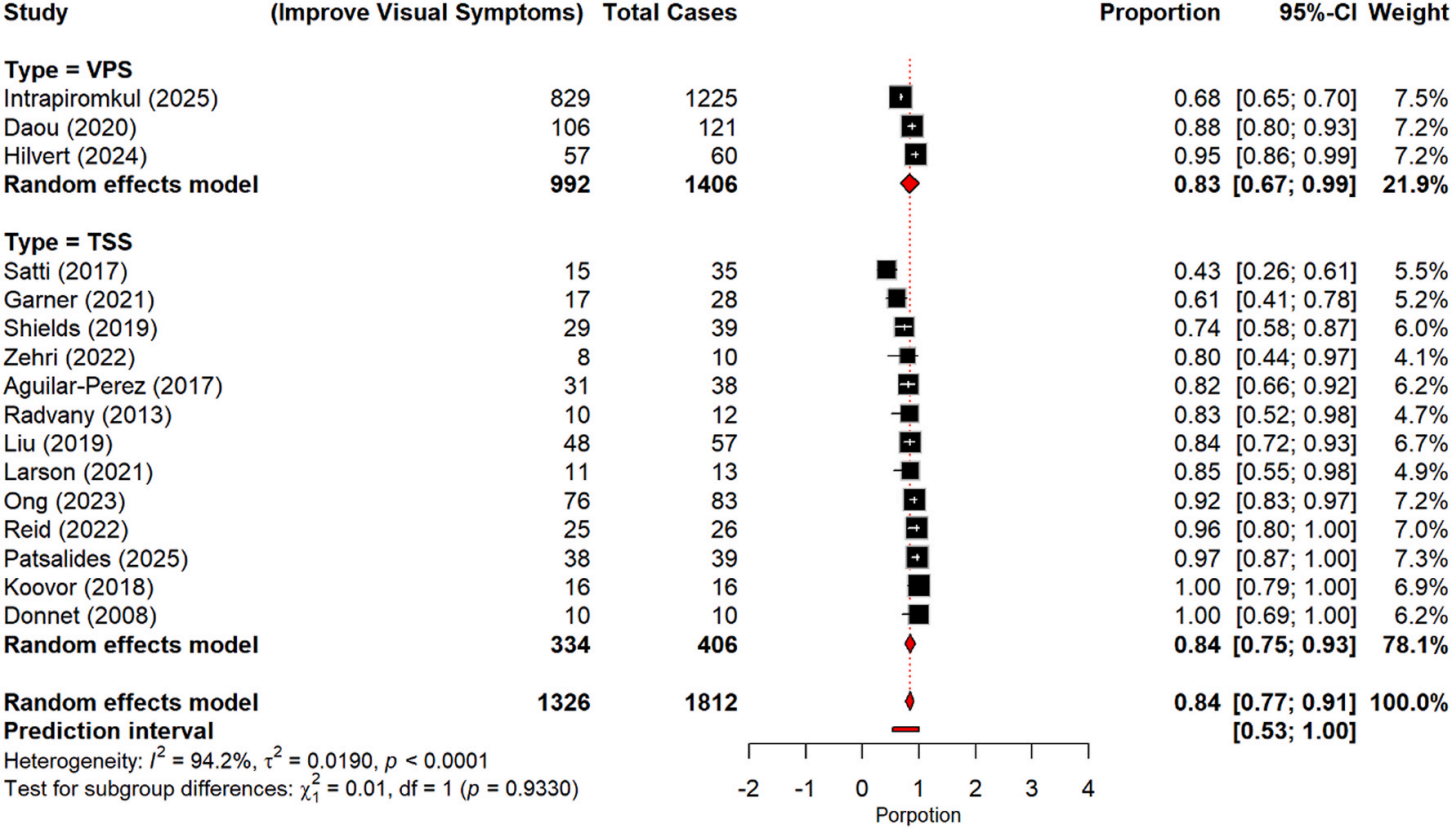
Comparison of Visual Symptoms Improvement between two Interventions.

Visual outcomes show high overall effectiveness across interventions. The pooled proportion of improvement is 83.9% (95% CI: 76.5%–91.4%), with no significant subgroup difference between TSS (84.1%) and VPS (83.3%) (p = 0.933). However, heterogeneity is extremely high (I^2^ = 94.2%), which showed variability across studies (Fig. 5).

**Fig. 6:**
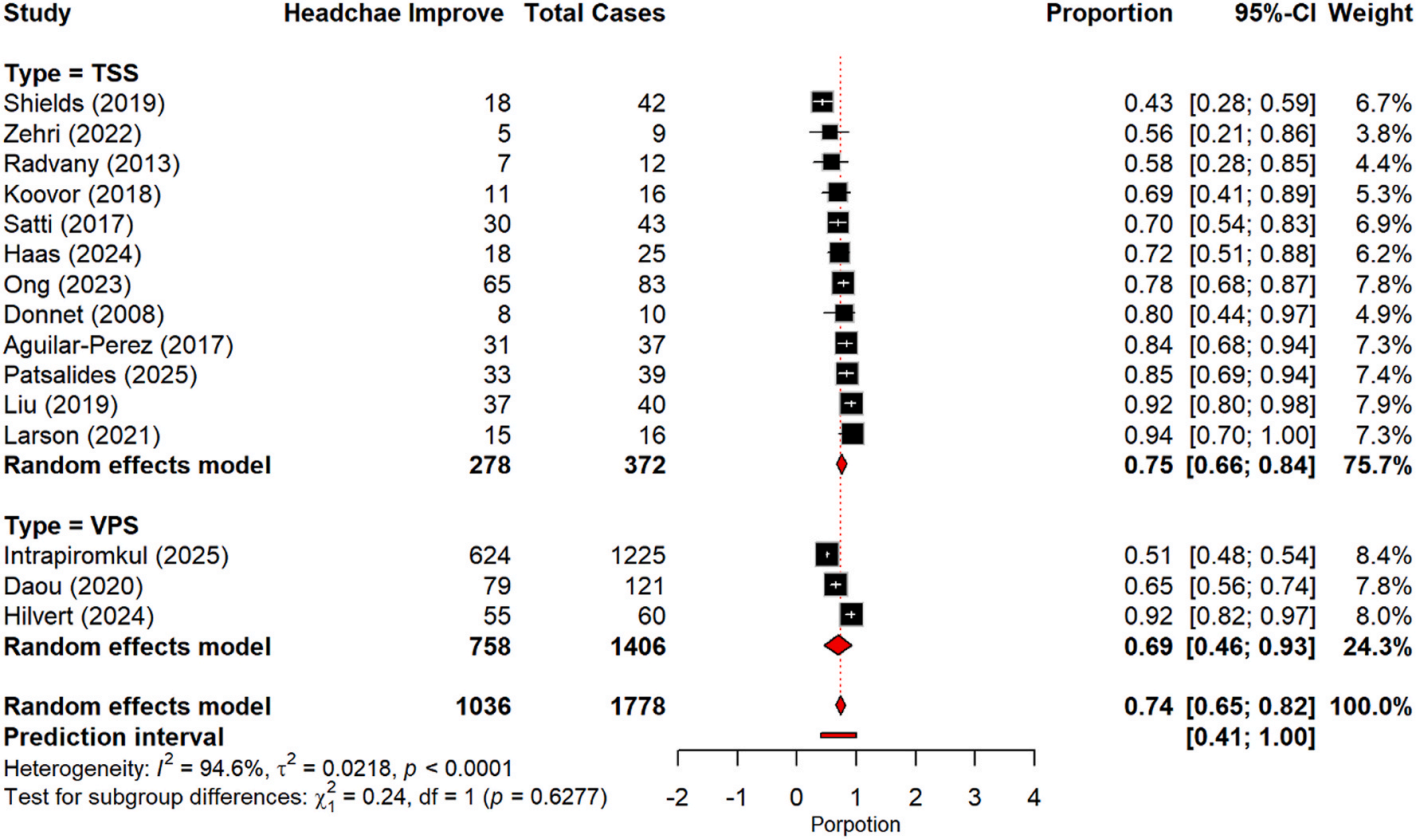
Comparison of Headache Improvement between two Interventions.

Notably, headache improvement follows a similar trend. The overall pooled improvement rate is 73.6% (95% CI: 65.2%–82.1%). TSS shows slightly higher improvement (75.4%) compared to VPS (69.2%), though this difference is not statistically significant (p = 0.628). Again, heterogeneity is considerable (I^2^ = 94.6%), which showed the subjective and multifactorial nature of headache in IIH (Fig. 6).

**Fig. 7:**
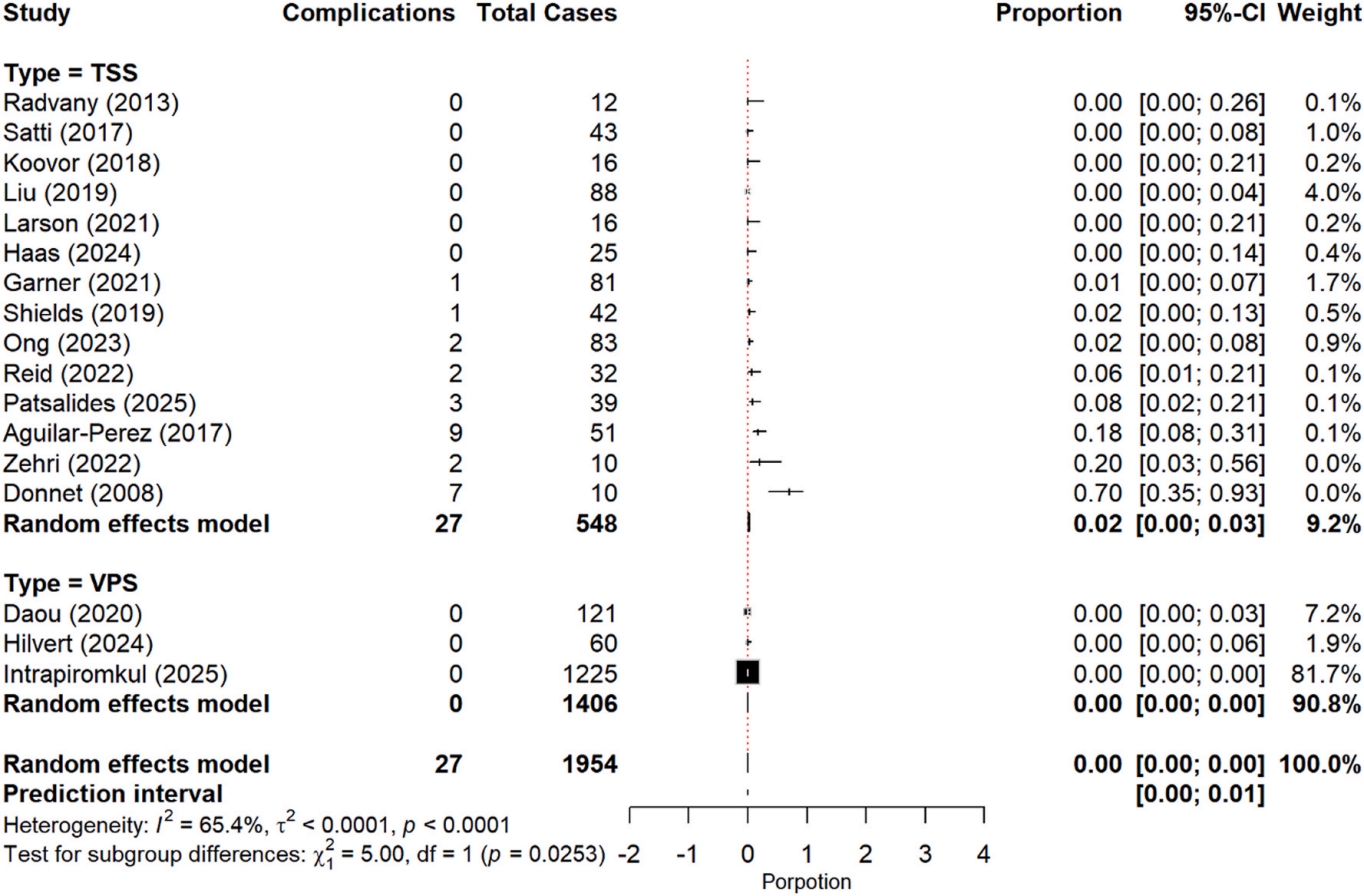
Comparison of Complication Rate between two Interventions.

Complication rates are low overall (0.1%; 95% CI: 0.0%–0.42%). Interestingly, subgroup analysis demonstrates that there is a statistically significant difference (p = 0.025), where TSS is reported to have a higher complication rate (1.56%) than VPS (approximately 0%). This is probably due to reporting bias and capture of small events in the TSS studies and not the actual procedural risk difference (Fig. 7). This finding contrasts with the neurosurgical literature, in which VPS is associated with a higher overall complication burden, and likely reflects inconsistent complication definitions across studies rather than a true difference in procedural risk.

**Fig. 8:**
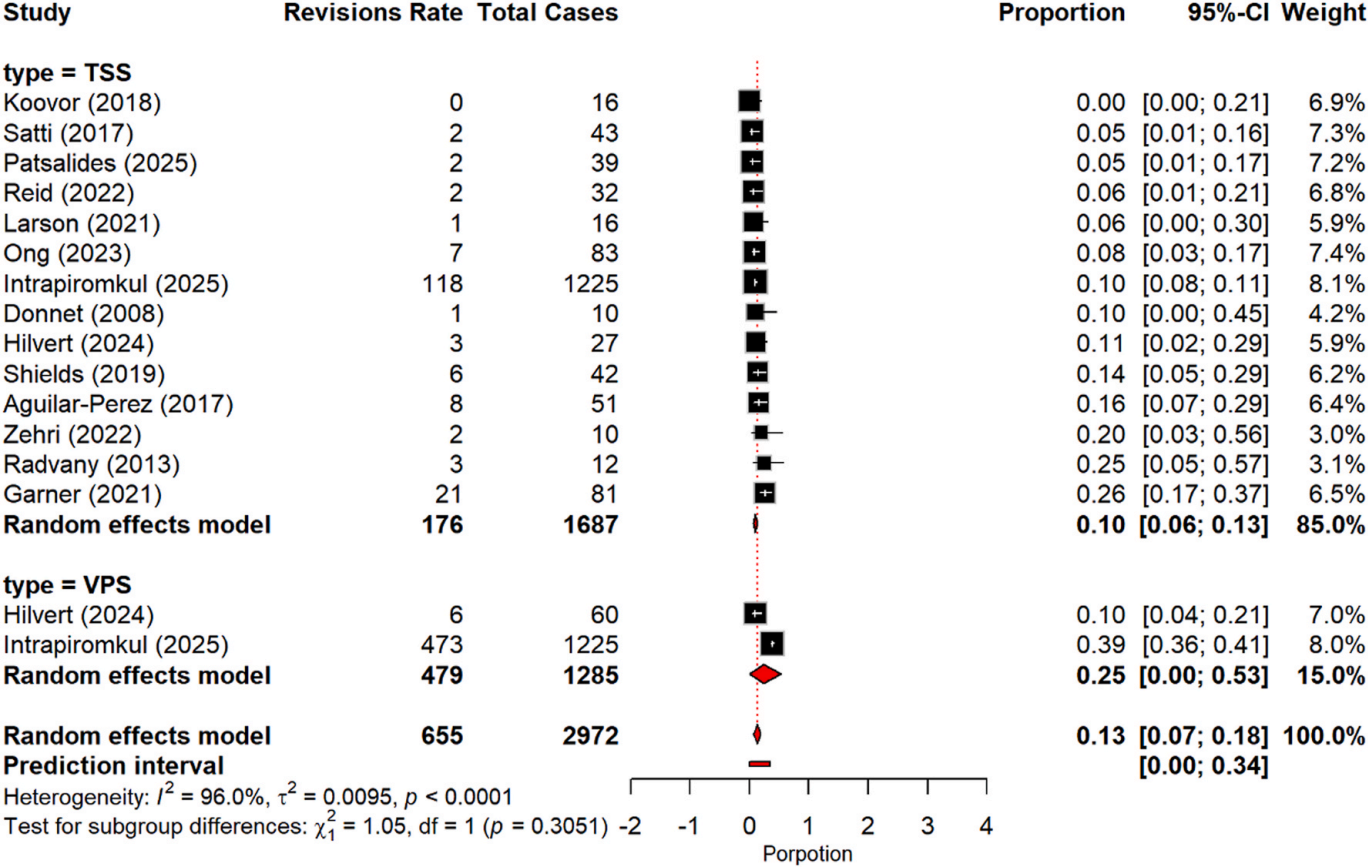
Comparison of Revision Rate between two Interventions.

Finally, revision rates favor TSS numerically. The pooled rate is 12.7% (95% CI: 7.3%–18.2%), with TSS at 9.8% versus VPS at 24.5%. Although this difference did not reach statistical significance (p = 0.305), these findings should be interpreted as a numerical trend rather than evidence of superior durability. (Fig. 8). A summarized comparison of pooled outcomes between TSS and VPS is presented in (Table 4).

**Table 4:** Comparative Summary of Important Findings (TSS vs VPS).

	TSS (Pooled Estimate)	VPS (Pooled Estimate)	Sig. Value (Subgroup)	I^2^ (%)
Intracranial Pressure (ICP)	SMD = −4.58	N/A	—	70.4
Visual Improvement	0.84	0.83	0.933	94.2
Headache Improvement	0.75	0.69	0.628	94.6
Complication Rate	0.016	0.000	0.025	65.4
Revision Rate	0.098	0.245	0.305	96.0

(Fig. 9) shows a mild asymmetry which suggested the possibility of publication bias. While several studies are distributed symmetrically around the pooled effect size, a noticeable clustering of points on the right side indicates a tendency toward the reporting positive outcomes. Furthermore, there are some smaller studies with more standard errors that seem scattered which added more to asymmetry. Nonetheless, most of the studies are within the confidence limits.

**Fig. 9:**
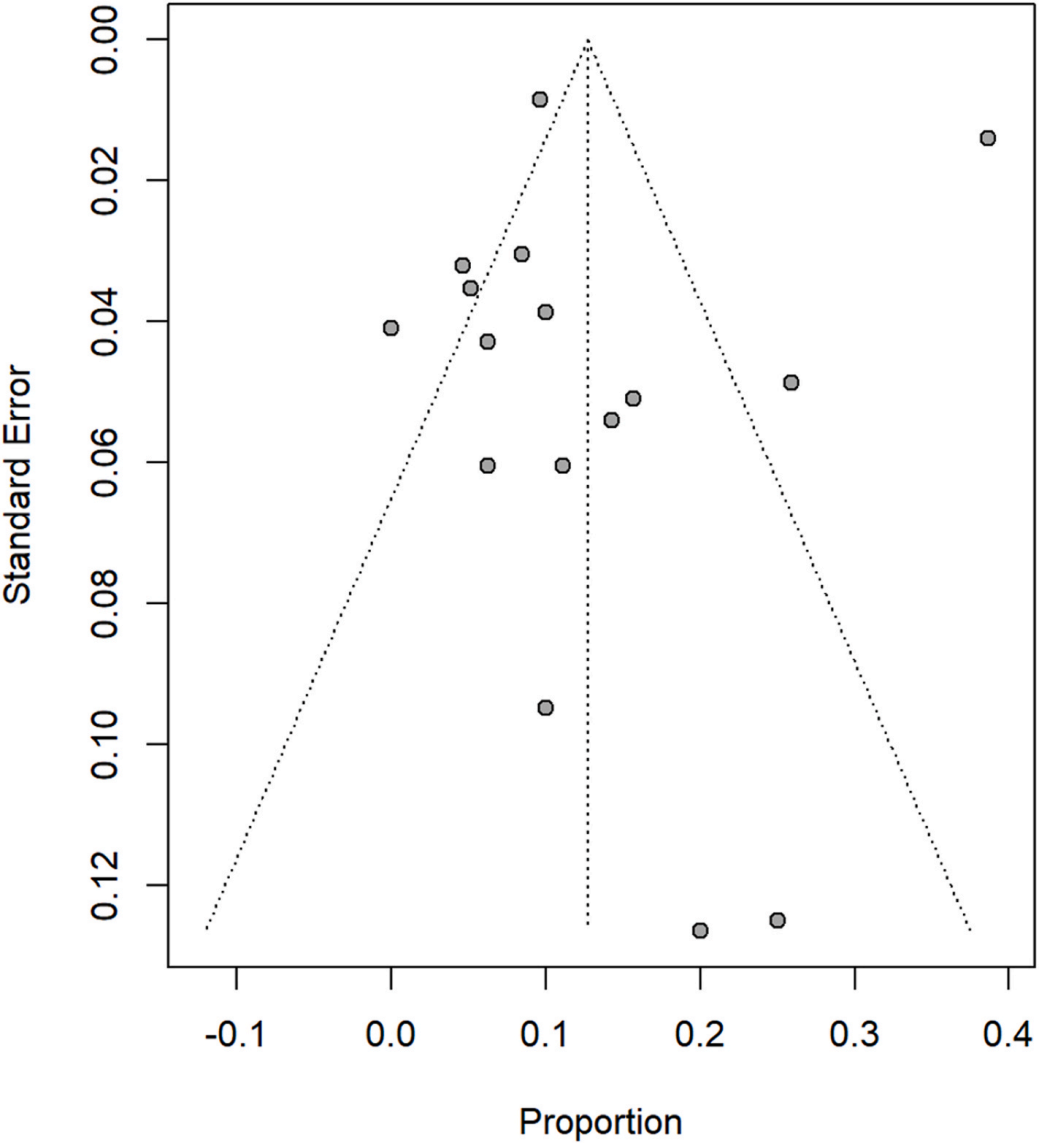
Assessment of publication bias with funnel plot.

## 4 Discussion

The present review provides a comprehensive synthesis of contemporary evidence comparing VPS and TSS in IIH, demonstrating that both interventions are effective in improving ICP, visual symptoms, and headache outcomes, with a consistent trend favoring TSS in terms of durability and lower re-intervention rates. These results are consistent with the recent literature, in which endovascular methods have become more clinically useful in the hands of carefully chosen patients. As an example, several modern reports have shown that venous sinus stenting is associated with substantial decreases in the CSF opening pressure and trans-stenotic gradients and high rates of symptom resolution, especially in patients who have a radiologically-confirmed venous sinus stenosis (Azzam et al., 2024; Fargen et al., 2023). Likewise, emerging prospective cohort evidence has solidified the idea that normalization of venous outflow is at the center of reversing the pathophysiology cycle of increased ICP, hence, the mechanistic explanation of TSS (Fargen, 2022). Conversely, although VPS is a well-established intervention with demonstrated effectiveness, its benefits are often offset by increased mechanical failure and chronic complications, which has contributed to an increasing questioning of its applicability as the first-line surgical modality in IIH treatment in the contemporary era (Andreão et al., 2024). It is important to emphasize that the ICP findings in this review reflect only within-group change following TSS, as no pooled VPS data were available for this outcome; therefore, no comparative or superiority claim regarding ICP reduction can be drawn between the two interventions from this analysis.

The similar enhancement in visual outcomes between TSS and VPS in the current study is notable especially because, in IIH, the main therapeutic objective is the preservation of vision. The last observations have continued to record improvement rates of more than 80% after venous sinus stenting with some groups showing almost full resolution of the papilledema in most of the patients (Azzam et al., 2024; Mansour et al., 2026). These results reflect our combined estimates and indicate that TSS can give visual results at least as good, or even a little better, than CSF diversion procedures. Moreover, the present comparative studies have shown that visual recovery after TSS can be faster because a direct hemodynamic correction of venous outflow obstruction takes place, and VPS is dependent on the slow diversion of the CSF and pressure (Handzic et al., 2025). Notably, new prospective studies have also focused on the contribution of underlying stenosis morphology intrinsic or extrinsic to the outcome of post-stenting visual outcomes as well, indicating that the patient-selection criteria could further maximize the effectiveness of the therapy (Tong et al., 2025). Taken together, these results support the increasing body that venous sinus pathology is no longer an epiphenomenon but a central therapeutic goal in IIH.

The results about headache, which are more variable and multifactorial, showed improvement in both intervention groups but not statistically significantly different, as is also observed in the literature. Recent reports have indicated the improvement rates of headache of 70-80% after venous sinus stenting, but they are not completely resolved because of the interplay of central sensitization, migraine overlap, and psychosocial factors (Oyemade et al., 2022). VPS has been linked to small headache relief, but the results are less predictable and may be affected by the complications of the shunts like over drainage or slit ventricle syndrome (Serra et al., 2025). Modern evidence indicates that although TSS is effective with regard to the venous aspect of intracranial hypertension, it may not be sufficient in overcoming headache syndromes, which are caused by non-pressure-related mechanisms, and hence the ongoing heterogeneity in studies (Lim et al., 2024). Moreover, recent findings suggest that adjunctive medical treatment could be necessary even after the intervention in a part of the patients, which proves the necessity of multimodal treatment methodology (Handzic et al., 2025).

A reduction in the rate of revision with TSS in comparison to VPS. It is important to note that this difference did not reach statistical significance; therefore, it should be interpreted as a numerical trend only, and not as confirmed evidence of superior mechanical durability for TSS over VPS. Recent cohort studies have proved that the rates of shunt revision may reach over 30-40% in the initial years of implantation mainly because of obstruction, infection, or mechanical failure, and the stenting procedures have relatively lower re-intervention rates (Shah et al., 2025). Furthermore, new evidence indicates that subsequent venous sinus stenting may cause major symptomatic reduction, even in those patients who already had shunts placed, and thus, it represents a potentially successful salvage therapy (El Naamani et al., 2023). It is also worth mentioning that restenosis and adjacent segment stenosis are also possible after TSS, and certain studies indicate that some patients should receive repeat stenting (Tanaka et al., 2025). However, the general profile of durability of TSS is positive, especially in comparison with long-term complications experienced with shunt dependency that is well documented.

The complication findings in this analysis must be interpreted with particular caution. Contrary to the broader neurosurgical literature which generally reports a higher complication burden for VPS due to infection, catheter malfunction, and over-drainage (Shah et al., 2025), our pooled analysis paradoxically found a statistically higher complication rate for TSS. We believe this most likely reflects differences in reporting depth and definitional inconsistency rather than a genuine safety disadvantage for TSS: endovascular literature has tended to report minor and access-related events more comprehensively, while several VPS studies, especially large retrospective cohorts, may have under captured delayed complications by classifying them as “revisions” rather than “complications.” Recent large series continue to show that serious TSS-related events, such as in-stent thrombosis or intracranial hemorrhage, are uncommon (Townsend et al., 2022), which is difficult to reconcile with the near-zero complication rate reported for VPS in our pooled estimate. Given this likely misclassification, our data should not be used to conclude that TSS carries a higher true procedural risk than VPS. Also, more recent research with a device focus has pointed to advances continuing in the design and methods of stent deployment that could continue to enhance safety in the future (Ramot et al., 2025). These advances note the significance of technological innovation within the context of IIH therapy.

Clinically, the results of this research have significant implications to treatment decision-making. The comparable efficacy and numerically lower revision rate observed with TSS suggest that it may be considered an appropriate first-line surgical option in carefully selected patients with demonstrable venous sinus stenosis and a significant trans-stenotic pressure gradient. Conversely, VPS remains an essential treatment option for patients without suitable venous anatomy, those requiring urgent cerebrospinal fluid diversion, or when endovascular treatment is not feasible. Moreover, the low invasiveness of TSS, as well as shorter hospitalization and quicker recovery duration, which have been described in the recent research, makes it more attractive in the contemporary clinical practice (Abbasi et al., 2024). Nevertheless, patient selection is very important and the chance of TSS success is strongly dependent on the existence of a remarkable trans-stenotic gradient of pressure and anatomical adequateness. On the other hand, VPS remains vital in patients with no venous sinus stenosis, or with rapidly progressive visual loss that needs urgent pressure relief. Thus, patient-centered care incorporating clinical, radiological, and hemodynamic values is needed to maximize the outcomes.

Beyond disease-specific and hemodynamic factors, real-world treatment selection between VPS and TSS is shaped by a set of practical and institutional considerations that are rarely captured in the included studies. Patients presenting with fulminant IIH and rapidly progressive visual loss often require urgent CSF diversion regardless of venous anatomy, given the immediate need for pressure reduction, whereas TSS candidacy is inherently restricted to patients with demonstrable venous sinus stenosis and a hemodynamically significant pressure gradient. In patients without significant stenosis, or with anatomy unsuitable for endovascular access, VPS remains the only viable surgical option. Beyond patient-specific anatomy, treatment choice is also strongly influenced by institutional availability of neurointerventional services, as TSS requires specialized endovascular expertise and infrastructure that may not be universally accessible, particularly outside high-volume tertiary centers. Surgeon and institutional experience with each technique further shapes practice patterns, introducing additional variability independent of patient factors. Recognizing these real-world determinants of treatment allocation reinforces that the comparative outcomes reported in this review should be interpreted as reflective of differently selected populations rather than as evidence of one intervention's inherent superiority over the other.

This study has several important limitations that should be carefully considered when interpreting the findings. First, the majority of included studies were non-randomized and retrospective in nature, introducing inherent risks of selection bias and confounding. Notably, treatment allocation was not random but strongly influenced by clinical, radiological, and institutional preferences**,** which creates systematic differences between patients undergoing VPS and those undergoing TSS**.**

Patients selected for TSS are typically those with demonstrable venous sinus stenosis and a measurable trans-stenotic pressure gradient, often after failure of medical therapy but in the absence of rapidly progressive visual decline. In contrast, VPS is frequently reserved for patients with more severe or fulminant disease**,** including those with acute visual deterioration, those without clear venous outflow obstruction, or those unsuitable for endovascular intervention. This indication bias likely results in fundamentally different baseline populations, thereby limiting the validity of direct comparisons between the two interventions.

Furthermore, the current literature often treats IIH as a homogeneous entity, whereas emerging evidence suggests that patients with true venous sinus stenosis associated with a hemodynamically significant pressure gradient may represent a distinct pathophysiological subgroup**.** These patients may respond preferentially to venous sinus stenting due to targeted correction of venous outflow obstruction. Conversely, patients without such gradients may have alternative mechanisms driving intracranial hypertension (e.g., CSF dysregulation), making them more suitable candidates for CSF diversion procedures. The lack of consistent reporting of venous pressure gradients, stenosis morphology (intrinsic vs extrinsic), and diagnostic thresholds across studies further limits subgroup-level interpretation.

In addition, there was substantial heterogeneity in study populations, diagnostic criteria, and outcome definitions, as reflected by high I^2^ values across multiple analyses. Variability in follow-up duration, procedural techniques, and outcome measurement tools also contributes to inconsistency in reported results. The scarcity of direct head-to-head comparative studies further complicates interpretation, as most studies evaluated VPS and TSS independently rather than within controlled comparative frameworks.

Importantly, this heterogeneity (I^2^ ranging from 65.4% to 96.0% across outcomes) was not further explored using meta-regression or pre-specified subgroup analyses, limiting our ability to statistically isolate clinically relevant effect modifiers. Follow-up duration varied markedly across studies (from 30 days to more than 60 months), so that short-term series may have systematically underrepresented late complications, restenosis, and revision events, while longer-term cohorts, though better positioned to capture durability, are more susceptible to loss to follow-up. Stent construct characteristics (length, diameter, number of stents deployed) were also inconsistently reported, despite their plausible relationship to adjacent segment stenosis and in-stent thrombosis risk. Likewise, the morphology of venous sinus stenosis intrinsic (e.g., arachnoid granulation hypertrophy, septae) versus extrinsic (e.g., compression from elevated ICP), was rarely characterized in the primary studies, despite evidence that this distinction may meaningfully influence stenting success and durability. The absence of meta-regression to formally test these covariates is an important methodological limitation and should temper confidence in the pooled effect estimates. Meta-regression was not performed because reporting of these study-level variables was inconsistent across the included studies, and only a limited number of studies provided sufficient data for robust statistical evaluation.

A related limitation stems from the structural composition of the evidence base itself. This review necessarily incorporated a mix of comparative cohort studies and non-comparative, single-arm case series, with pooled VPS and TSS estimates frequently derived from studies examining only one intervention rather than both within the same clinical setting. This approach relies on an indirect, across-study comparison rather than direct within-study contrasts, diluting comparative validity because the two intervention arms are drawn from different institutional protocols, surgeon preferences, diagnostic thresholds, and patient populations rather than a shared clinical environment. Consequently, apparent differences between TSS and VPS in this analysis may partly reflect between-study or between-center variation rather than a true treatment effect, and this indirectness should be considered when interpreting the pooled comparative estimates.

Another important limitation concerns the extraction and interpretation of complication data. Our pooled analysis found a paradoxically low, near-zero complication rate for VPS relative to TSS, a finding discordant with the broader neurosurgical literature, in which VPS is generally reported to carry a higher overall complication burden, commonly cited in the range of 2–5% or more when hardware failure, infection, and revision-related events are considered (Maroufi et al., 2026), compared with TSS. This discrepancy most likely reflects heterogeneous and inconsistent definitions of “complication” across the included studies rather than a genuine safety advantage for VPS. Many VPS studies, particularly larger retrospective cohorts such as Intrapiromkul et al. (2025) (Intrapiromkul et al., 2025), may have reported only immediate periprocedural adverse events while classifying delayed shunt malfunctions, infections, or obstructions as “revisions” rather than “complications,” effectively removing these clinically significant events from the complication rate. Retrospective database studies relying on administrative or ICD-10 coding are additionally prone to undercapturing complications managed as outpatient events or coded under revision procedures (Dao Trong et al., 2023). Given this likely misclassification, the complication comparison reported here should be interpreted with considerable caution and should not be taken to imply that TSS carries a higher true procedural risk than VPS; if anything, established comparative literature suggests the opposite. Future studies should adopt standardized, prospectively defined complication taxonomies (e.g., distinguishing periprocedural, delayed, and revision-related events) to permit valid safety comparisons between these interventions.

Finally, reporting bias and publication bias remain potential concerns, particularly given the tendency for newer endovascular studies to report favorable outcomes. Long-term durability data, especially for TSS in relation to restenosis and need for re-stenting, are still limited and inconsistently reported.

The future studies should aim at overcoming these shortcomings by conducting well designed prospective studies and randomized controlled trials that will directly compare TSS and VPS among homogeneous patients. Prospective, phenotype-guided trial designs exemplified by the recently reported RIVER study (Patsalides et al., 2025), represent an important model for how future comparative research in this field should be structured, enrolling patients according to standardized hemodynamic and radiological criteria and applying consistent, prospectively defined outcome and complication definitions. The long-term follow-up data are especially required to determine the sustainability of venous sinus stenting and risk of restenosis in the long run. Moreover, more research on patient selection criteria, such as the contribution of pressure gradient thresholds and imaging biomarkers, can also be used to narrow down indications of TSS. The further development of the device technology, as well as the creation of stents that are specifically oriented towards the intracranial venous anatomy, are equally subjects to further investigation. Patient-reported outcomes and quality of life measures should be integrated into future research to fully understand the potential effect of such interventions on patient well-being and inform evidence-based clinical practice.

## 5 Conclusion

This review demonstrates that both VPS and TSS are effective in managing IIH, with comparable improvements in visual outcomes and headache symptoms, and a large reduction in ICP following TSS for which no comparative VPS data were available. TSS showed a numerically lower revision rate than VPS (9.8% vs. 24.5%), but this difference was not statistically significant; this should be interpreted as a potential trend rather than demonstrated evidence of superior durability, and warrants confirmation in adequately powered comparative studies. These conclusions, however, must be weighed against the profound indication bias inherent in the underlying literature: because no included study randomly allocated patients to VPS or TSS, the comparative estimates reported here reflect differences between fundamentally distinct clinical populations rather than a true head-to-head treatment effect. As the two interventions are typically applied to different patient populations. TSS is most often performed in patients with demonstrable venous sinus stenosis and a trans-stenotic pressure gradient, whereas VPS is frequently reserved for more severe or non-venous cases. These observations support the concept that IIH may represent a heterogeneous spectrum**,** in which patients with true venous sinus stenosis and a pressure gradient may constitute a distinct subgroup that could be more appropriately classified as a venous outflow–related form of intracranial hypertension. Overall, treatment decisions should be individualized based on underlying pathophysiology, and future studies should focus on standardized patient selection and direct comparative designs to better define optimal management strategies.

## Declaration of interests

The authors declare that they have no known competing financial interests or personal relationships that could have appeared to influence the work reported in this paper. No external funding, grants, or industry support was received for the conduction of the study, analysis, or preparation of the manuscript. The authors have not received honoraria, consulting fees, stocks, or other forms of compensation from entities with an interest in the subject matter discussed. All authors had full access to the data and take responsibility for the integrity and accuracy of the analysis. The manuscript has not been published previously and is not under consideration elsewhere.
